# Efficacy of paracetamol, diclofenac and advice for acute low back pain in general practice: design of a randomized controlled trial (PACE Plus)

**DOI:** 10.1186/s12891-017-1432-5

**Published:** 2017-02-01

**Authors:** M. Schreijenberg, P. A. J. Luijsterburg, Y. D. M. Van Trier, D. Rizopoulos, M. A. Koopmanschap, L. Voogt, C. G. Maher, B. W. Koes

**Affiliations:** 1 Department of General Practice, Erasmus MC, University Medical Center, PO box 2040, 3000 CA Rotterdam, The Netherlands; 2 Department of Biostatistics, Erasmus MC, University Medical Center, PO box 2040, 3000 CA Rotterdam, The Netherlands; 3 Department of Health Policy and Management/iMTA, Erasmus University Rotterdam, PO Box 1738, 3000 DR Rotterdam, The Netherlands; 4 Dutch Association for Back pain Patients ‘The Spine’, Bentinckstraat 21, Lichtenvoorde, The Netherlands; 5 The George Institute for Global Health, University of Sydney, PO Box M201, Sydney, NSW 2050 Australia

**Keywords:** Low back pain, Therapy, General practitioners, General practice, Pain, Analgesics, Acetaminophen, Anti-inflammatory agents, Non-steroidal, Diclofenac, Randomized controlled trial, Clinical trial

## Abstract

**Background:**

Low back pain is common and associated with a considerable burden to patients and society. There is uncertainty regarding the relative benefit of paracetamol and diclofenac and regarding the additional effect of pain medication compared with advice only in patients with acute low back pain. This trial will assess the effectiveness of paracetamol, diclofenac and placebo for acute low back pain over a period of 4 weeks. Furthermore, this trial will assess the additional effectiveness of paracetamol, diclofenac and placebo compared with advice only for acute low back pain over a period of 4 weeks.

**Methods:**

The PACE Plus trial is a multi-center, placebo-blinded, superiority randomized controlled trial in primary care, with a follow-up of 12 weeks. Patients with acute low back pain aged 18–60 years presenting in general practice will be included.

Patients are randomized into four groups: 1) Advice only (usual care conforming with the clinical guideline of the Dutch College of General Practitioners); 2) Advice and paracetamol; 3) Advice and diclofenac; 4) Advice and placebo. The primary outcome is low back pain intensity measured with a numerical rating scale (0–10). Secondary outcomes include compliance to treatment, disability, perceived recovery, costs, adverse reactions, satisfaction, sleep quality, co-interventions and adequacy of blinding.

Between group differences for low back pain intensity will be evaluated using a repeated measurements analysis with linear effects models. An economic evaluation will be performed using a cost-effectiveness analysis with low back pain intensity and a cost-utility analysis with quality of life. Explorative analyses will be performed to assess effect modification by predefined variables.

Ethical approval has been granted. Trial results will be released to an appropriate peer-viewed journal.

**Discussion:**

This paper presents the design of the PACE Plus trial: a multi-center, placebo-blinded, superiority randomized controlled trial in primary care that will assess the effectiveness of advice only, paracetamol, diclofenac and placebo for acute low back pain.

**Trial registration:**

Dutch Trial Registration NTR6089, registered September 14th, 2016. Protocol: Version 4, June 2016.

## Background

Low back pain is one of the most common diseases of the musculoskeletal system. It is associated with a considerable burden to patients and society. According to the global burden of disease study, low back pain is the number one disorder responsible for disability in the population (as calculated by the years lived with disability (YLD)) [1]. The point prevalence is reported to be as high as 33%. The total costs associated with back pain in The Netherlands are estimated at 3,5 billion euro in 2007 [2]. In the United States, the figure is over US$50 billion [3].

Clinical guidelines for the management of low back pain have been issued in many countries around the world in order to promote rational care [4]. These guidelines provide clear agreement on the recommendations for first line care of acute low back pain [4]. According to most guidelines, first line care should consist of reassurance on the favorable prognosis of non-specific low back pain, advice to stay active and avoid bed rest, and prescription of a simple analgesic medicine using a time-contingent dose regimen, e.g. 1 g paracetamol administered 4 times per day. The clinical guideline for the management of low back pain of the Dutch College of General Practitioners (NHG) also recommends paracetamol as first choice followed by nonsteroidal anti-inflammatory drugs (NSAIDs) as a second option for the prescription of analgesics for patients with acute low back pain [5]. The current guideline preference for paracetamol as the first choice analgesic was not based on evidence on its efficacy in patients with back pain, but on its better safety-profile as compared to NSAIDs and other analgesics. Until recently there was no placebo-controlled trial available evaluating the effect of paracetamol for patients with low back pain.

In July 2014, the first placebo controlled trial of paracetamol for acute low back pain (PACE trial) was published [6]. Australian researchers showed no difference in clinical outcomes between paracetamol and placebo in patients with acute low back pain. In this large clinical trial, 1652 patients with acute low back pain were randomized to (1) paracetamol on regular doses (2) paracetamol as needed or (3) placebo. Neither on the primary outcome (time to recovery) nor on any secondary outcome such as back pain intensity, disability, symptom change were differences in outcome between the three study groups found [6].

Considering the findings in this Randomized-Controlled Trial (RCT), one relevant question is if the current clinical guideline recommendations should be changed regarding the use of paracetamol. The Australian research team stated that replication of their study findings should take place before dismissing paracetamol as a treatment option for low back pain. Changing the content of guidelines based on the findings of a single trial without verification of the results in other similar populations would seem premature [7]. Besides replication of the paracetamol versus placebo contrast of the PACE trial two other topics are also of importance: firstly, there is ample evidence that the clinical course of many patients with acute low back pain is rather favorable. In the PACE trial the median recovery was 16–17 days in all participating patients, including those receiving placebo, and by 12 weeks about 85% of patients was recovered. All patients in the trial received advice and reassurance of a favorable prognosis in addition to the study medications and apparently did rather well regarding the authors. This raises the question of whether patients with acute low back pain need paracetamol (or other analgesic) at all. What would be the outcome if patients receive advice and reassurance only?

Secondly, the awareness of the limited clinical effect of paracetamol could easily influence the decision to step up more quickly to using NSAIDs which are the next recommended type of pain medication in the clinical guidelines. Should NSAIDs even be recommended as first analgesic treatment option instead of paracetamol for patients with acute low back pain? NSAIDs have been compared with placebo in patients with low back pain and have shown significantly better results for pain reduction [8]. However, the magnitudes of the effects are rather small. The between-group differences were less than 10 points on a 0–100 pain scale. In addition, in direct comparisons NSAIDs have not shown consistent superiority above paracetamol in patients with acute low back pain. The Cochrane review only lists 5 RCTs comparing NSAIDS versus paracetamol and all were at risk for high risk of bias [8]. The Cochrane review concluded ‘whether NSAIDs are more effective than other drugs or non-drug therapies for acute low-back pain still remains unclear’. In the Netherlands, diclofenac has been the most commonly prescribed NSAID over the past decade [9].

### Objective

The primary objective of the PACE Plus trial is to compare the clinical effectiveness of paracetamol, diclofenac and placebo for acute low back pain in primary care over 4 weeks of follow-up. Furthermore, this trial aims to determine the added clinical effectiveness of medication and advice (paracetamol, NSAID or placebo) versus advice only for acute low back pain in primary care over 4 weeks of follow-up. Secondary objectives of the PACE Plus trial are to compare disability, patients’ perceived recovery, quality of life, costs, time to recovery, compliance to treatment, adverse reactions, patients’ satisfaction, sleep quality and co-interventions between advice plus paracetamol, advice plus diclofenac, advice plus placebo and advice only groups.

## Methods/design

### Trial design and setting

**Fig. 1:**
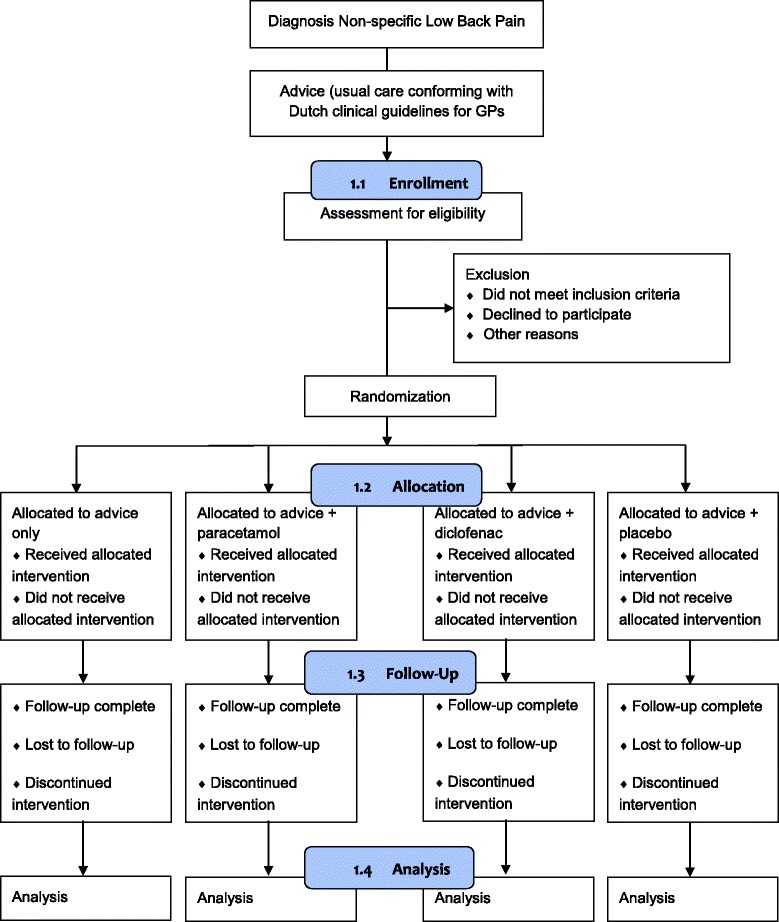
Flow-chart of the PACE Plus trial

The trial will be a four arm, multicenter, placebo-blinded, superiority randomized controlled trial using double dummy technique in general practice with a follow-up period of 12 weeks. The study design and flow of patients in the PACE Plus trial are shown in Fig. 1.

The patient eligibility criteria of the PACE trial are similar to the in- and exclusion criteria that will be used in the PACE Plus trial. Based on the figures in the PACE trial, we will need to assess at least 2231 patients to end up with 800 patients that fulfill eligibility criteria and are willing to participate in the trial. In the PACE trial, 4606 patients were screened by 235 primary care providers during a recruitment period of 3.5 years. This comes down to an average of 5.6 patients per primary care provider per year. The PACE Plus trial has a planned recruitment period of 2 years. We thus need cooperation of at least 200 General Practitioners (GPs) for the referral of patients with acute low back pain for screening. Based on the Dutch National Assessment of Diseases in Primary Care [10], in the average Dutch general practice, the incidence of low back pain is 27 per 1000 patients per year; we therefore assume the proposed referral rate is feasible. Recruitment rate will be monitored closely during the trial recruitment period and if necessary, more GPs will be contacted for participation.

### Participants and eligibility criteria

Patients will be recruited in Dutch general practices and referred to the PACE Plus research team. Before enrolment in the trial, all potential patients will be assessed for eligibility and informed consent.

### Inclusion criteria

In order to be eligible to participate in this study, a patient must meet all of the following criteria: 1) Aged between 18 and 60 years; 2) Low back pain of less than 6 weeks duration; 3) Primary complaint of pain in the area between the 12th rib and buttock crease, with or without radiating leg pain; 4) Experiencing a new episode of low back pain, preceded by a period of at least 1 month without low back pain; 5) Low back pain severe enough to cause at least moderate pain (≥4 on 0–10 numerical rating scale (NRS)).

### Exclusion criteria

A potential patient who meets any of the following criteria will be excluded from participation in this study: 1) known or suspected serious spinal pathology (e.g. metastatic, inflammatory or infective diseases of the spine, cauda equina syndrome, spinal fracture); 2) Currently taking recommended regular doses of analgesics, including paracetamol or diclofenac; 3) Spinal surgery within the preceding 6 months; 4) Serious co-morbidities like severe rheumatoid arthritis, cardiac failure, diabetes preventing prescription of paracetamol (e.g.: liver or renal failure) or diclofenac (e.g. gastric ulcers or other gastro-intestinal problems); use of proton pump inhibitors before inclusion is not an exclusion criterium, as the patient is considered to be protected (patient will have to continue using this medication during use of study medication); 5) Use of coumarine derivatives, clopidogrel, prasugrel, ticagrelor, acetylsalicylacid derivatives, systemic glucocorticoid, selective serotonin reuptake inhibitors (SSRIs), venlafaxine, duloxetine, trazodone, spironolactone or other medications that may interact with paracetamol and/or diclofenac; 6) Known intolerance for paracetamol and/or diclofenac; 7) Pregnant or planning to become pregnant during the treatment period.

### Recruitment

Patients consulting their GP or doctor’s assistant for low back pain and fulfilling simple referral criteria (ages 18 to 60 years, new episode of low back pain (6 weeks maximum duration) and no contraindications for diclofenac) can be referred to the PACE Plus research team. Potential participants will be contacted within 24 h by a researcher for further information about the trial, assessment of the eligibility criteria and collection of informed consent.

### Randomization and blinding

After collection of informed consent, patients will be randomly allocated to one of four intervention groups: 1 advice only group and 3 medication groups. Randomization will be performed using a two-step process. In the first step, patients will be randomized between ‘advice only’ and ‘medication’ using a computer-generated randomization list. After the first step of the randomization process, patients and GPs will be informed about the outcome of treatment allocation (either that they receive advice only or that they receive blinded study medication).

In the advice only group, patients will not get study medication, but receive advice and reassurance from their GP or doctor’s assistant only (usual care conforming with the clinical guideline of the Dutch College of GPs).

For people who are randomized in the first step to ‘medication’, a trial medication prescription will be sent to the Erasmus University Hospital Trial Pharmacy. In the second step of randomization, an independent trial pharmacist will use a randomization list with random blocks to determine the medication group that patients will be randomized to (paracetamol, diclofenac or placebo). Both randomization lists used in this two-step process are made by an independent data-manager who is not involved in this trial.

After allocation to 1 of the 3 medication groups, patients will receive a treatment pack containing large oblong tablets and small round tablets prepared and numbered by an independent trial pharmacist. Treatment packs will be sent by mail to the patient, and are expected to arrive the next day. Using the double dummy technique, active medication differs between groups as follows:

- Paracetamol group: active oblong tablets (active paracetamol) and placebo round tablets (placebo diclofenac);
- Diclofenac group: placebo oblong tablets (placebo paracetamol) and active round tablets (active diclofenac);
- Placebo group: placebo oblong tablets (placebo paracetamol) and placebo round tablets (placebo diclofenac).

The placebo tablets that will be used in the PACE Plus trial are identical in appearance and taste to their active counterparts, but do not contain the active component. All medication packaging will be identical between the 3 medication groups, except for a unique randomization number for each participant. Every package contains a reply paid post envelope, in which unused tablets can be returned for counting after 4 weeks of follow-up. The patient, patient’s GP and pharmacist and researchers involved in data collection and analysis will be blind to treatment group allocation. Unblinding is permissible in case of a reported suspected unexpected serious adverse reaction (SUSAR).

### Treatment

All patients in the PACE Plus trial will receive advice and reassurance from either their GP or doctor’s assistant before referral (usual care conforming with the clinical guideline of the Dutch College of GPs).

Patients in the medication groups will be asked to take 4 daily doses of 2 oblong tablets and 2 daily doses of 1 round tablet, until they have experienced two consecutive pain free days (NRS 0 or 1 out of 10), or for a maximum of 4 weeks if a pain free interval does not occur. This means that treatment groups will receive the following drug dosages:

- Paracetamol group: paracetamol (immediate release) 4 daily doses of 1000 mg, placebo diclofenac 2 daily doses.
- Diclofenac group: diclofenac (immediate release) 2 daily doses of 75 mg, placebo paracetamol 4 daily doses.
- Placebo group: placebo paracetamol 4 daily doses, placebo diclofenac 2 daily doses.

Allocated treatment as described above may be discontinued by the patient’s own GP in case the patient revisits his or her GP because of persisting low back pain; this will be recorded during follow-up measurements.

### Co-interventions

During participation in the PACE Plus trial, patients in the medication groups will be asked not to take paracetamol or NSAIDs because this may lead to overdose of these medications. Participant’s GP and Pharmacist will be informed about the participation of their patient in the PACE Plus trial, and for the medication groups, the usage of trial medication. Additional medication taken by the patient for low back pain will systematically be recorded in patients’ questionnaires at all follow-up measurements. Physiotherapy as a co-intervention is allowed, but will also be recorded in follow-up measurements.

### Outcomes

The primary outcome of the PACE Plus trial is low back pain intensity measured with an 11-point NRS (score range 0–10; higher score means more pain). Pain intensity will be recorded daily over a 4 week follow up period.

Secondary outcome measures that are collected in the PACE Plus trial are:

- compliance to treatment measured daily by asking ‘How many large, oblong tablets did you take today?’ and ‘How many small, round tablets did you take today?’ (questions derived from the Brief Medication Questionnaire (BMQ) [11].)
- disability measured using the Roland Morris Disability Questionnaire (RMDQ; score range 0–24; higher score means more disability) [12].
- patients’ perceived recovery measured using a 7-point Likert scale that will be dichotomized into recovered (score 1 ‘complete recovery’ and 2 ‘much improved) and not-recovered (score 3 ‘improvement’ to score 7 ‘worse than ever’).
- quality of life measured using the EuroQol Group 5 Dimensions, 5 Level Questionnaire (EQ-5D-5 L) [13].
- costs; all direct medical and patient costs measured using the iMedical Consumption Questionnaire (iMCQ), and productivity costs measured with iProductivity Cost Questionnaire (iPCQ) [14, 15].
- time to recovery assessed using the daily low back pain severity scores. Recovery is defined as the first day of 0 or 1 pain intensity, maintained for seven consecutive days.
- adverse reactions systematically recorded in the follow-up questionnaires; all reported adverse events will be followed until they have abated or until a stable situation has been reached.
- patients’ satisfaction measured using an 11-point NRS; score range 0–10, higher score means more satisfaction.
- sleep quality measured using a 4 point Likert scale derived from the Pittsburgh Sleep Quality Index (PSQI) [16]. Scores will be dichotomized into good sleep quality (score 1 ‘very good’ and 2 ‘fairly good’) and poor sleep quality (score 3 ‘fairly bad’ and 4 ‘very bad’).
- co-interventions systematically recorded in the follow-up questionnaires.
- adequacy of blinding assessed in medication groups by asking patients to which treatment group they believe to be allocated after 12 weeks of follow-up.

Baseline characteristics that will be measured in the PACE Plus trial (including potentially relevant prognostic factors) are:

- gender, age, height, weight, education and occupational status.
- duration of complaints, history of back complaints, and comorbidity.
- job satisfaction measured with a 7-point Likert scale (score range from extremely unsatisfied to extremely satisfied).
- neuropathic pain measured with the Pain DETECT questionnaire (score range 0–38; higher score means a neuropathic component of back pain is more likely) [17].
- potentially modifiable prognostic indicators measured with the StarT Back Tool [18].

### Patient timeline and data collection

Table 1 shows the time schedule of patient enrollment, interventions and assessments according to the SPIRIT-statement [19]. After collection of informed consent, patients will fill out the baseline questionnaire. Subsequently, patients will be randomized into one of the four treatment groups. Patients will fill out daily digital questions regarding low back pain severity and compliance to treatment during 4 weeks after baseline measurement. Questionnaires concerning secondary outcomes will be filled out at 2, 4 and 12 weeks of follow-up. All questionnaires used in the PACE Plus trial will be sent to participants using e-mail and filled out using secure hyperlinks. If a questionnaire is not filled out (completely) by a participant, the research team will send a reminder encouraging the participant to complete the questionnaire.

**Table 1:**
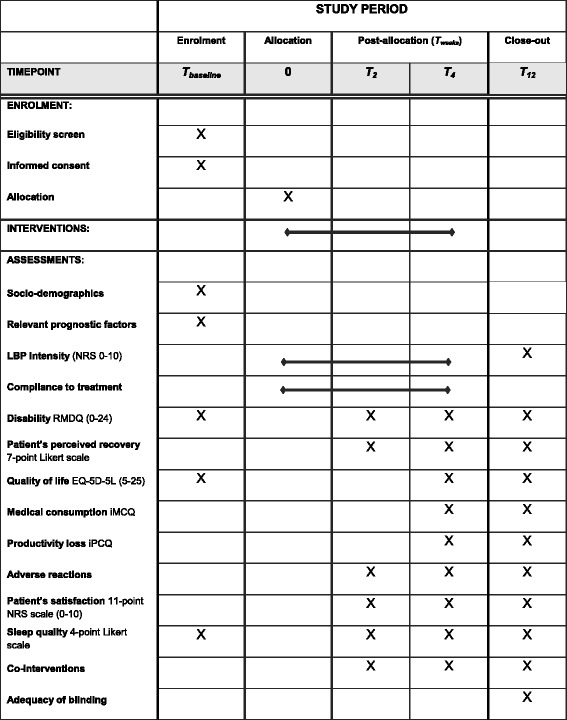
Schedule of enrolment, interventions and assessments (SPIRIT)

*LBP* low back pain,*NRS* numerical rating scale score, *RDMQ* roland-morris disability questionnaire, *EQ-5D-5 L* EuroQol Group, 5 dimensions, 5 level questionnaire, *iMCQ* institute for Medical Technology Assessment (iMTA) medical consumption questionnaire, *iPCQ* iMTA productivity cost questionnaire

### Sample size

For the primary outcome (low back pain intensity (NRS)), between group differences of at least 20% are considered clinically relevant; this difference is expressed in the area under the longitudinal pain trajectories for the four treatment groups. Because low back pain is an episodic condition that is known to fluctuate over time, the correlation between repeated measured was assumed moderate (the parameter rho of a first-order auto-regressive serial correlation structure was set to 0.7). In the sample size calculation, a statistical power of 84% and a random dropout not exceeding 15% were assumed. With group sizes of 200 patients, a between group difference in low back pain intensity of at least 20% can be detected.

### Statistical analysis

The statistical analysis will be performed according to the intention-to treat principle.

### Primary statistical analysis

For clinical effectiveness the between group differences for the primary outcome, low back pain-intensity will be evaluated using a repeated measurements analysis with linear mixed effects models with adequate specification of the fixed and random effects structures to account for possible nonlinear effects. The covariance structure will be unstructured, but we will compare Akaikes’ information criterion between the different covariance structures and choose the structure with the lowest value.

### Secondary statistical analysis

A similar approach as described in ‘primary study parameter(s)’ will be used for the continuous secondary outcomes (e.g. disability and quality of life) to assess between group differences.

A Cox proportional hazards model will be carried out to evaluate the difference in time to recovery (recovery is defined as seven consecutive low back pain NRS scores of 0–1) between the groups.

The effect modification of the allocated treatment strategy by predefined baseline variables (explorative) on low back pain intensity, disability and recovery at 4 and 12 weeks follow-up will be analysed by Cox proportional hazard analyses and logistic regression analyses, respectively. Predefined variables are severe low back pain (defined as NRS ≥ 7) and severe disability (defined as RMDQ ≥ 16) at baseline.

To assess the cost-effectiveness of paracetamol versus diclofenac versus advice only for acute low back pain in general practice, a cost-effectiveness analysis will be performed using the primary outcome low back pain severity (measured daily). A cost-utility analysis will be performed to compare our study with other studies in musculoskeletal disorders research in a more general accepted outcome e.g. quality of life (measured in Quality-Adjusted Life Years (QALYs)). Utility values of the Dutch public for EuroQol health states will be applied to calculate QALY’s based on the EQ-5D. Using non parametric bootstrapping (randomly drawing 2500 observations with replacement from the patient sample), the degree of uncertainty for costs and health effects and the cost-utility ratio will be depicted in a cost-effectiveness plane. In addition, an acceptability curve will be drawn, which indicates the probability that the paracetamol or diclofenac versus advice only has lower incremental costs per QALY gained than various thresholds for the maximum willingness to pay for an extra QALY.

The economic analysis will be based on the societal perspective and on the healthcare perspective in which the direct and productivity costs in the groups will be compared. The costs per hour of productivity loss will be updated from the Dutch Guideline for economic evaluations in health care [20]. The friction cost method will be used to calculate the productivity costs according to the Dutch guidelines. The costs per unit of medical consumption will be estimated, using information from the Dutch Manual for economic evaluation of health care on costs per unit of medical services [21].

### Data management and safety

All personal data (e.g. demographics, contact-data, questionnaires, diary) will be stored anonymously. The patients’ identity will remain confidential at all times. Each patient will be allocated a unique code, which will be used on the Case Report Forms (CRFs). The link between the code and the patients’ name will only be assessed by the researchers and the data-manager.

Trial conduct and data integrity will be audited once per year by independent auditors.

## Discussion

This paper presents the design for a randomized, placebo controlled trial that will assess the effectiveness of paracetamol, diclofenac and placebo for acute low back pain in primary care. Furthermore, the trial will assess the additional effectiveness of paracetamol, diclofenac and placebo compared to advice only for acute low back pain in primary care. The primary outcome is low back pain intensity measured daily on a numerical rating scale over a period of 4 weeks. Secondary outcomes are measured at 1 weeks, 2 weeks, 4 weeks and 12 weeks of follow-up and include compliance to treatment, disability, perceived recovery, costs, adverse reactions, satisfaction, sleep quality, co-interventions and adequacy of blinding. Between group differences for the primary outcome will be evaluated using a repeated measurements analysis with linear effects models. An economic evaluation will be performed using a cost-effectiveness analysis with low back pain intensity and a cost-utility analysis with quality of life. Explorative analyses will be performed to assess effect modification by predefined variables. The outcomes of this trial may impact the clinical guideline recommendations concerning first analgesic treatment options in acute low back pain in general practice.

Recruitment of eligible patients is currently ongoing. Substantial protocol amendments will be communicated to participants, cooperating GPs and pharmacists, Medical Research and Ethics Committee (MREC), the Dutch Trial Registry, ZonMw and the journal publishing this protocol. Results of this trial will be published in a peer-reviewed journal. After publication, participating patients and GPs will be informed about trial results (expected in 2020).

## Abbreviations

BMQ: Brief medication questionnaire
CRF: Case report form
EQ-5D-5 L: EuroQol group, 5 dimensions, 5 Level questionnaire
GP: General practitioner
iMCQ: iMTA medical consumption questionnaire
iMTA: Institute for medical technology assessment
iPCQ: iMTA productivity cost questionnaire
LBP: Low back pain
MREC: Medical research and ethics committee
NHG: Dutch College of general practitioners (Nederlands Huisartsen Genootschap)
NRS: Numerical rating scale
NSAID: Nonsteroidal anti-inflammatory drug
NTR: Dutch trial register (Nederlands Trial Register)
PSQI: Pittsburgh sleep quality index
QALY: Quality-adjusted life years
RCT: Randomized controlled trial
RMDQ: Roland Morris disability questionnaire
SSRI: Selective serotonin reuptake inhibitor
SUSAR: Suspected unexpected serious adverse reaction
YLD: Years lived with disability
ZonMw: Netherlands Organization for Health Research and Development
